# Oscillating Structural Transformations in the Electrochemical Synthesis of Graphene Oxide from Graphite

**DOI:** 10.1002/anie.202411673

**Published:** 2024-10-18

**Authors:** Bartosz Gurzęda, Paweł Jeżowski, Nicolas Boulanger, Alexandr V. Talyzin

**Affiliations:** ^1^ Department of Physics Umeå University Umeå S-90187 Sweden; ^2^ Institute of Chemistry and Technical Electrochemistry Poznan University of Technology Berdychowo 4 Poznan 60-965 Poland

**Keywords:** Oscillations, Graphene Oxide, Electrochemical Oxidation, Synchrotron Radiation, X-ray Diffraction

## Abstract

Electrochemical synthesis of graphene oxide (GO) is known to occur with potential oscillations, but the structural changes underlying these oscillations have remained unclear. In situ time‐resolved synchrotron radiation X‐ray diffraction demonstrates that the electrochemical synthesis of GO in aqueous H_2_SO_4_ can be described as an oscillating reaction. The transformation from graphite to GO proceeds through periodic structural oscillations that correlate with potential cycles. Stage‐1 graphite intercalation compound (GIC) is found only at the peak of the potential cycle, but not at the bottom of the cycle. Stage‐1 GIC is formed in the first half‐cycle from stage‐2 GIC and then transforms into “pristine graphite oxide” (PGO) on the lower side of the potential cycle, after which the cycle restarts with the formation of a new portion of stage‐1 GIC. Water‐washing results in the transformation of PGO into water‐swollen GO with d(001) ~11 Å. These periodic structural changes can be considered a new type of oscillating reaction. The presented results provide broad insights into the oscillating structural changes occurring during the anodic graphite oxidation in aqueous H_2_SO_4_ and allow to update of the mechanism of GO electrochemical formation.

## Graphical abstract

In situ time‐resolved X‐ray diffraction studies of electrochemical graphite oxidation in aqueous sulfuric acid reveal periodic structural changes of a graphite intercalation compound (GIC) into “pristine graphite oxide”. The potential oscillations of the graphite electrode correlate with the appearance and disappearance of a solid phase identified as stage‐1 GIC. The observed phenomenon can be considered as a new type of oscillating reaction.

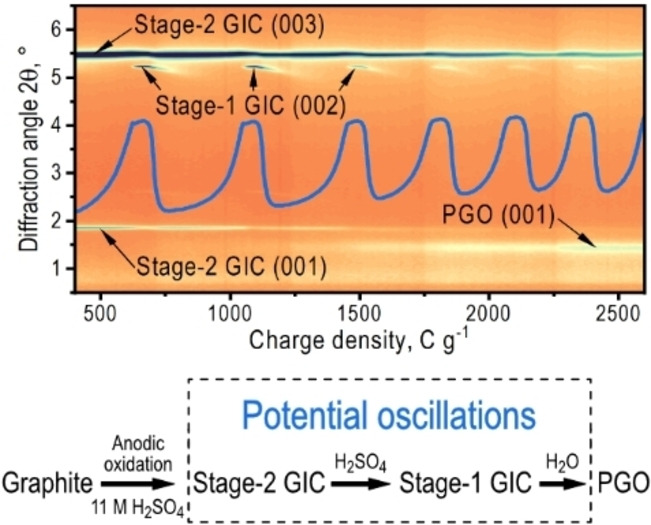

## Introduction

Graphite oxides (GO) are materials produced by the strong oxidation of graphite and exfoliated to produce single‐layered graphene oxide. Enormous interest in graphene oxides over the last 15 years was initially motivated by the possibility of converting it into graphene.^[1]^ In more recent years, the interest has shifted towards applications that do not require reduction, for example, in various types of membranes,[^2^, ^3^, ^4^, ^5^] sorbents for toxic gases,^[6]^ water contaminants^[7]^ or isotopic selectivity,^[8]^ biomedicine,^[9]^ composites,^[10]^ etc.

Most commonly, GO is synthesized by a solution route using a strong acid, which expands the graphitic lattice by intercalation, allowing a strong oxidant to penetrate interlayer spaces.[^11^, ^12^, ^13^, ^14^, ^15^] The reaction of the oxidant with graphene layers results in functionalization with a variety of oxygen functional groups.^[16]^ However, using strong oxidants and concentrated acids is environmentally unfriendly and requires elevated safety precautions.[^17^, ^18^, ^19^]

An alternative way to synthesize GO is electrochemical oxidation of graphite. In recent years, interest in the preparation of GO by electrochemical methods has increased significantly due to environmental friendliness, safety, ease of control, and potentially low cost. The entire synthesis is carried out at ambient temperature with no oxidants and with the amounts of reagents significantly reduced.[^19^, ^20^, ^21^, ^22^]

The anodic oxidation of graphite to GO can be performed in aqueous inorganic acid solutions such as H_2_SO_4_,[^20^, ^22^, ^23^, ^24^] HClO_4_,[^21^, ^25^] HNO_3_,[^26^, ^27^] or HBF_4_.^[28]^ Graphite is first intercalated by the acid during the electrochemical oxidation, followed by co‐intercalation of water molecules, which react with positively charged graphene layers, resulting in the addition of surface oxygen functional groups to the graphene layers.[^24^, ^25^] It is well known that the intercalation of acid into the graphite structure occurs with the formation of graphite intercalation compound (GIC) as an intermediate product.

Moreover, electrochemical oxidation of graphite under conditions of constant applied current occurs with potential oscillations observed for the first time by Krohn et al. in 1982 in 12 M H_2_SO_4_. This rare and intriguing oscillation mechanism of graphite oxidation has been very little studied. The exact structures formed in the process of oxidation at each stage of the potential cycle remain unknown until now. The only attempt of in situ study available in the literature dates back to 1992, with technical limitations related to conventional X‐ray diffraction (XRD) methods that did not allow for rapid time‐resolved data recording and were not sufficiently sensitive to detect low‐intensity signals.^[29]^ The study reported the formation of stage‐2 GIC at the maximum of potential oscillation, and a weak reflection at the potential minimum, which was assigned to stage‐3 GIC (stage‐*n* is defined as the *n* number of graphene layers between neighboring intercalated layers). A few years later, Beck et al. proposed the zone model of electrochemical GO formation based on galvanostatic graphite oxidation in a well‐defined range of aqueous H_2_SO_4_ concentrations.^[24]^ The model suggests that water molecules co‐intercalate into the graphitic lattice after the formation of stage‐2 GIC and react with graphene layers forming C−OH groups during the increasing part of the potential cycle. Further oxidation of C−OH groups into C=O was assumed to happen during the decreasing part of the potential cycle. However, more recent studies indicated that the decrease in potential in each cycle is associated with water molecules co‐intercalation into GIC interlayers, and the increase in potential is likely associated to the formation of oxygen functional groups on graphene layer surface.^[26]^ Surprisingly, despite extensive studies of the electrochemical synthesis of GO performed over the past decades, the origin of potential oscillations and the mechanism of GO formation remained unclear.

Structural studies performed in situ directly during the process of electrochemical oxidation are required to reveal the fundamental mechanism of graphite electrooxidation. The lack of in situ studies is likely related to technical issues. Limitations are provided by the relatively complex electrochemical cell encapsulating graphite immersed in strong acids, rapidly changing structures during the cycles, and the relatively long time required to achieve full oxidation. However, modern synchrotron radiation XRD methods provide the possibility of relatively rapid data recording in combination with powerful radiation suitable for in situ studies of liquid‐immersed and encapsulated samples.

Here we report an in situ time‐resolved XRD study of an electrochemical reaction which leads to the transformation of graphite into “pristine graphite oxide“ (PGO) and further to the formation of “standard” GO typically studied in ex situ experiments after water‐washing. A microscopic capillary‐sized electrochemical reactor was designed and successfully implemented to identify all the intermediate phases formed within cycles of potential oscillations, leading to the formation of GO in H_2_SO_4_. Our experiments revealed rather unusual periodic structural changes, in several ways similar to oscillating chemical reactions[^30^, ^31^, ^32^] which are typically related to changes in the concentration of reaction components in solutions. However, the case of periodic appearance and disappearance of solid phases detected by XRD in our study is rather unusual and can be described as a new type of oscillating reaction with a previously unexpected complexity of electrochemical graphite oxidation.

## Results and Discussion

**Figure 1:**
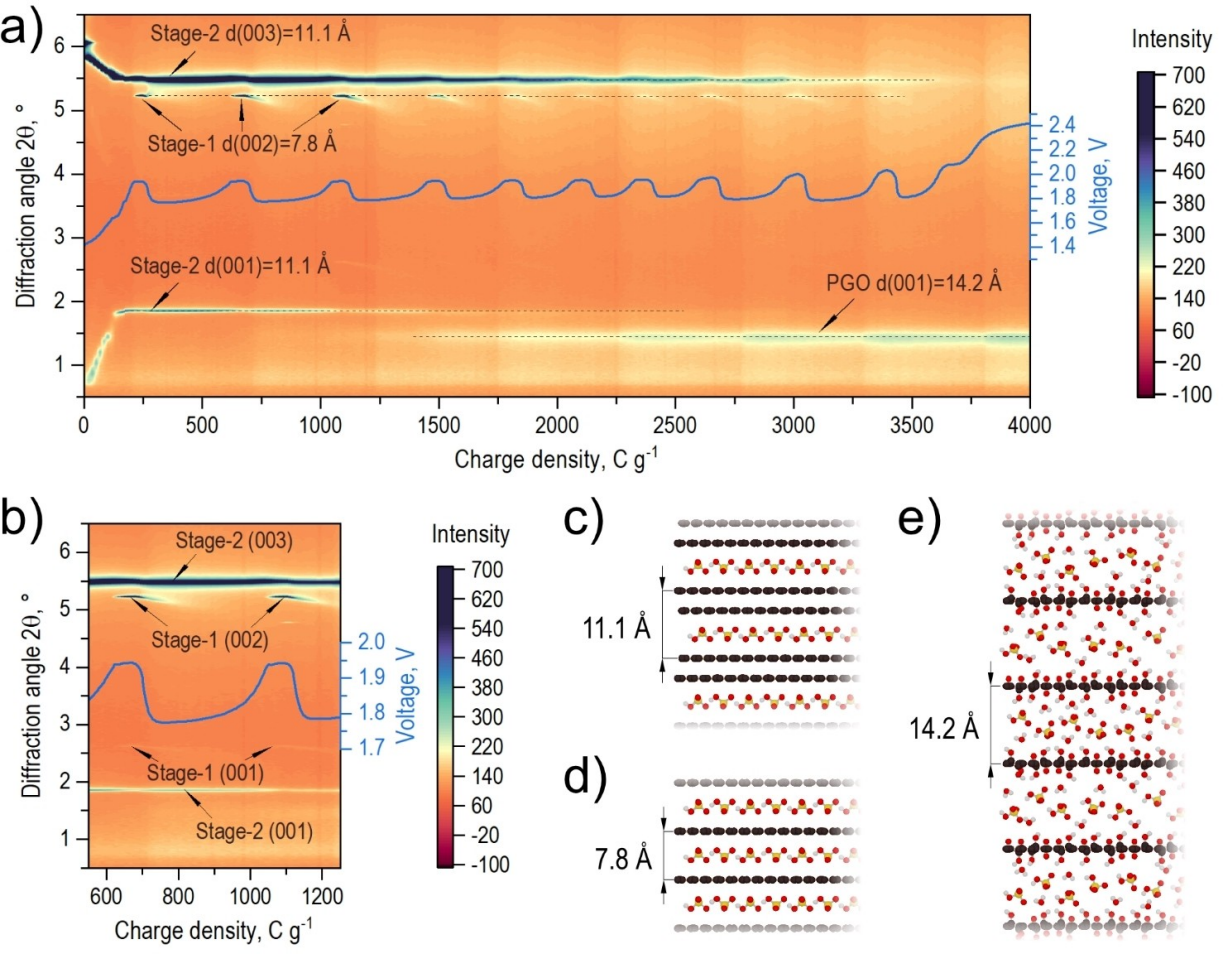
a) “Heat map” constructed using XRD patterns (λ=0.35435 Å) recorded during the electrochemical graphite oxidation combined with the galvanostatic curve showing voltage oscillations, b) enlarged region of the first cycle showing correlation between the changes of sample structure and voltage. Schematic structures of phases revealed by XRD: graphite structures intercalated by H_2_SO_4,_ c) stage‐2 GIC and d) stage‐1 GIC. e) The structure of electrochemically synthesized PGO.

Electrochemical oxidation of graphite was studied here using a specially designed microscopic reactor assembled inside a glass Pasteur pipette. The cell allows simultaneous recording of electrochemical and XRD data. As expected, the galvanostatic curve recorded in this microscopic electrochemical reactor showed voltage oscillations during the electrochemical oxidation of graphite in 11 M H_2_SO_4_ under conditions of constant applied anodic current (Figure 1a and b). The shape of the galvanostatic curve is nearly the same as the curve recorded in the three‐electrode system for bulk graphite electrooxidation (Figure S1) indicating that the observed voltage oscillations are caused by the potential oscillations of the graphite electrode. Using synchrotron radiation, we were able to record XRD images every minute and identify all crystalline phases which appeared (and disappeared) during one cycle (48 min for the first cycle). Phase transformations inside the reactor were also monitored over a period of several hours until oscillations finally faded due to the nearly complete conversion of graphite into PGO.

As expected, simple immersion of graphite into H_2_SO_4_ did not result in any structural changes. The data collected with the constant applied current show an initial rise of voltage with rapid changes in the sample structure and a prolonged region of voltage oscillations correlated with periodic structural changes (Figure 1a). The galvanostatic curve is plotted here as a function of charge density delivered to the working electrode (see Figure S3 for the same curve plotted as a function of time). Prior to the start of voltage oscillations, graphite, positively charged by constant anodic current, undergoes rapid structural changes due to sulfuric acid intercalation into the interlayer spaces and the formation of GIC. Analysis of XRD data recorded during the initial rise of voltage shows a set of structural transformations related to the formation of GIC with progressively larger amounts of intercalated acid (Figure S4). Additional reflections assigned to the stage‐6, stage‐4, and stage‐3 were found in XRD patterns when the working electrode acquired charge density equal to 52, 68, and 84 C g^−1^, respectively (Figure S5). When the charge density of 120 C g^−1^ is achieved to the working electrode, the stage‐3 GIC starts to transform into the stage‐2 GIC, which is reflected by a small voltage plateau at 1.65 V in the galvanostatic curve. Finally, at the charge density of 200 C g^−1^, the XRD patterns show only the (00 *l*) reflections assigned to pure stage‐2 H_2_SO_4_‐GIC with a *d‐*spacing of 11.1 Å (Figure 1c and Figure S6).^[33]^

Periodic potential oscillations at the working electrode were observed after the voltage increased to 1.94 V. These oscillations, with a period of 31–49 mins (Figure S3), were recorded over several hours with XRD data continuously collected every minute. As mentioned above, complete conversion of graphite into stage‐2 H_2_SO_4_‐GIC was detected by XRD already prior to the start of oscillations. According to the earlier published models (mostly based on ex situ experiments), a gradual step‐like transformation of stage‐2 GIC into PGO could be expected during the reaction.[^24^, ^26^] However, analysis of collected data revealed significantly more complex processes.

Surprisingly, XRD detected the stage‐1 H_2_SO_4_‐GIC with an interlayer spacing of 7.8 Å[^16^, ^19^] formed at the first maximum of voltage (Figure 1d and Figure S6). This phase was never previously observed in relation to electrochemical oxidation of graphite and potential oscillations. Figure 1 shows that voltage suddenly drops after a part of the stage‐2 H_2_SO_4_‐GIC is transformed into the stage‐1 GIC. Unexpectedly, the intensity of (002) reflection assigned to the stage‐1 GIC decreases during the voltage drop until it disappears completely. The voltage stabilizes at the low point of the cycle at 1.76 V and after ~200 C g^−1^ starts increasing again. According to previously proposed models, the second part of cycle, with an increase of voltage, was assigned to the water co‐intercalation between the graphite interlayer spaces occupied by the intercalate and the reaction between the H_2_O and graphene layers resulting in the formation of GO.^[26]^ However, the formation of GO as a result of potential oscillations was never previously related to the stage‐1 GIC found in our experiments. Figure 1 illustrates an intriguing observation of periodic appearance and disappearance of this phase correlated with the voltage oscillations.

**Figure 2:**
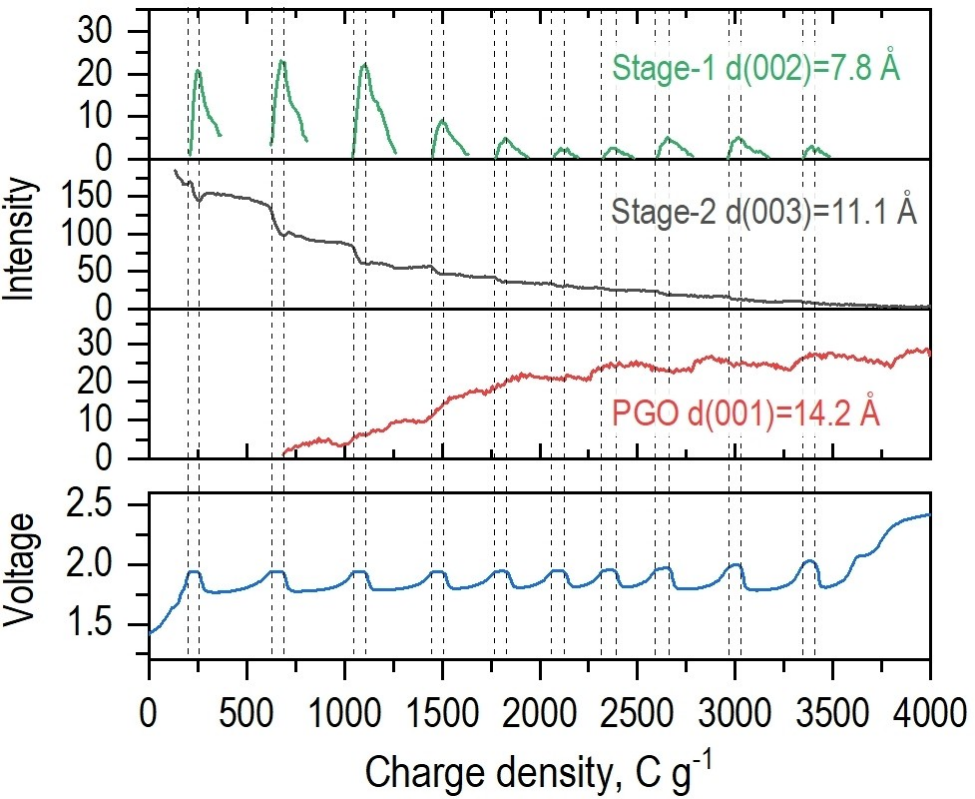
The changes in the intensities of signals assigned to stage‐1 GIC, stage‐2 GIC and PGO caused by the electrochemical graphite oxidation.

When all the stage‐1 GIC formed during the first part of the cycle was completely converted into PGO and disappeared, the voltage increased again up to 1.94 V and a new cycle started. The data presented in Figures 1 and 2 show how the XRD reflections from the stage‐1 GIC appear and disappear periodically in correlation with the increase and decrease of the voltage in all cycles of oscillations. Simultaneously, the intensity of (001) reflection of PGO increases until the voltage oscillations cease, indicating almost complete transformation of graphite into PGO.

Overall, the electrochemical oxidation of graphite revealed in our experiments can be described as following:

– The stage‐2 GIC is formed at the first stage of the reaction. However, this phase is not directly converted into PGO.
– A small part of the stage‐2 GIC is converted into the intermediate stage‐1 GIC at the top plateau of the voltage cycle but disappears at the bottom part of the cycle.
– The stage‐1 GIC is converted into “pristine GO” (PGO) and completely consumed before the start of a new cycle. “Pristine” is used here for GO prior to water‐washing in order to distinguish it from “standard” GO studied in ex situ experiments after water‐washing.
– The new portion of the stage‐1 GIC is then formed from the stage‐2 GIC in the next cycle and is again transformed into PGO.

The periodic nature of the electrochemical oxidation of graphite is highly unusual, possibly representing a new type of oscillating reaction. Unlike other oscillating reactions related to changes in the concentrations of reaction components in liquid solutions,[^30^, ^31^, ^32^] the electrochemical oxidation of graphite goes through periodic appearance and disappearance of a solid phase (stage‐1 GIC) with a well‐defined structure.

The hypothetical reaction model proposed above can be further confirmed by the analysis of the intensities of XRD reflections from the different phases over the course of the reaction. The evolution of the stage‐1 (002) and stage‐2 (003) intensities (Figure 2) demonstrates correlated changes indicating the exact time point when stage‐2 GIC is converted into stage‐1 GIC. The step‐like increase of intensity of (001) reflection is evident also for PGO, which correlates with the drop in intensity and disappearance of stage‐1 (002). Therefore, the periodic changes in the intensity of the three main phases demonstrate that each voltage cycle is caused by the formation of stage‐1 GIC from stage‐2 GIC followed in the next step by water co‐intercalation and conversion of stage‐1 GIC into PGO. The changes in half‐width at half maximum (HWHM) of the (00 *l*) reflections assigned to stage‐1 (002), stage‐2 (003), and PGO (001) also correlate with the observed periodic structure changes (Figure S7). Just before the stage‐1 GIC is formed, the HWHM of stage‐2 (003) reflection increases slightly, indicating the insertion of H_2_SO_4_ into the GIC matrix. The HWHM of stage‐1 (002) reflection increases clearly due to H_2_O co‐intercalation and stage‐1 GIC transformation into PGO. With each observed oscillation, the HWHM of PGO (001) reflection progressively decreases and at the same time the HWHM of stage‐2 (003) reflection progressively increases, indicating a gradual electrochemical transformation of GIC into GO.

A new weak reflection with an interlayer distance of ~14.2 Å assigned to PGO is evident already in the second cycle after ~600 C g^−1^. The intensity of PGO reflections increases over the time of reaction (Figure 1e and Figure S6). Finally, when the voltage oscillations fade after prolonged reaction (~3700 C g^−1^), the voltage increases to 2.4 V indicating the electrolysis of water.[^24^, ^26^] No further structural changes were observed after the end of oscillations. We note that a weak reflection due to stage‐2 GIC is still present at the end of the reaction, indicating incomplete conversion into PGO.

**Figure 3:**
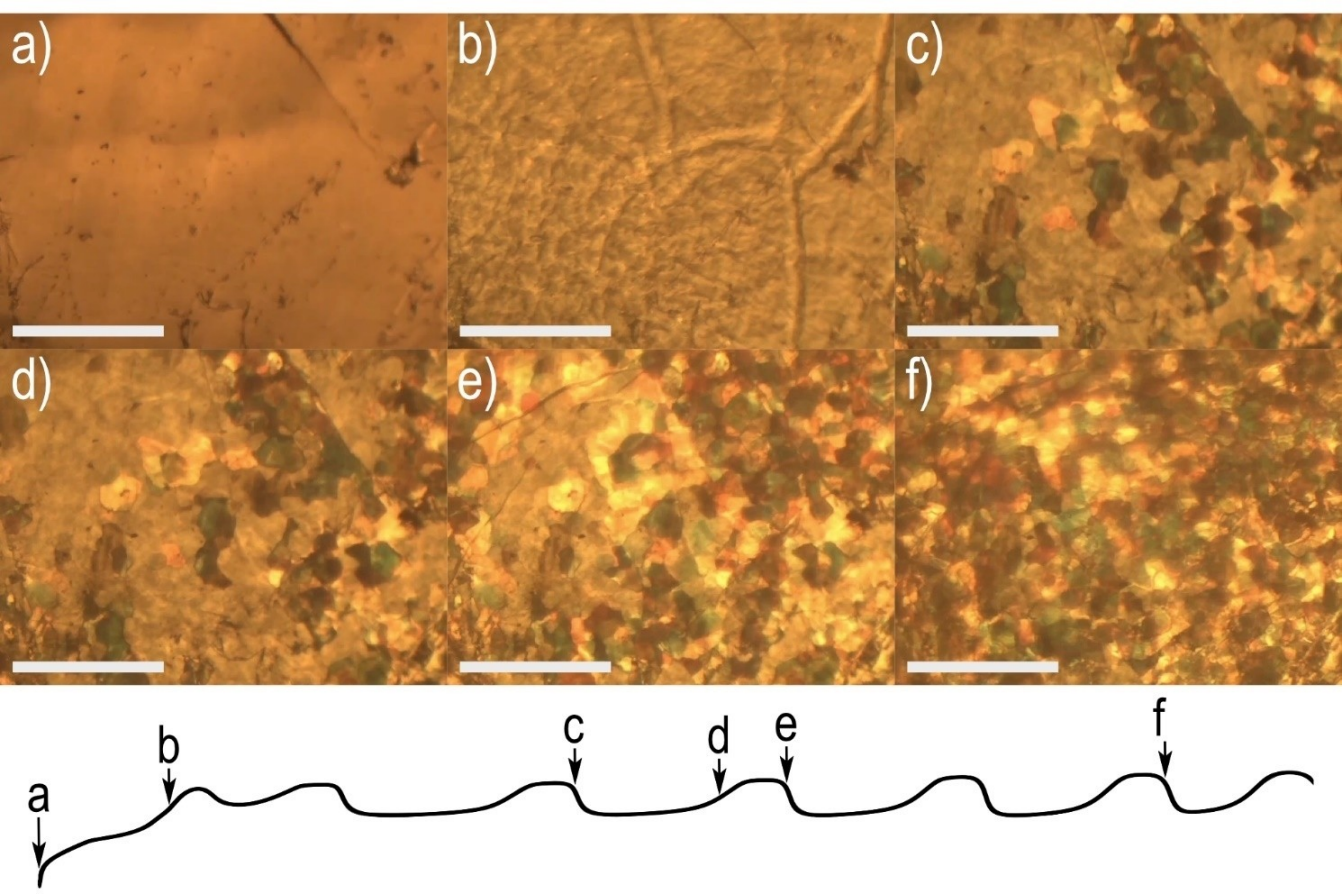
In situ optical microscope observations of graphite transition into PGO correlated with specific points in the galvanostatic curve. a) Smooth graphite surface immersed in 11 M H_2_SO_4_ prior to oxidation. b) Wrinkled stage‐2 GIC formed during the anodic oxidation of graphite. c–f) Images of intermediate phases composed of stage‐2 GIC, stage‐1 GIC and PGO. Scalebars: 100 μm.

Periodic changes in the sample structure revealed by XRD are also confirmed by visual observations using optical microscopy (Figure 3 and Video S1). Highly oriented pyrolytic graphite was used in this experiment and a video was recorded over the period of several voltage oscillations under conditions of electrochemical oxidation in 11 M H_2_SO_4_.The formation of stage‐2 GIC during an initial increase of voltage is clearly visible due to the expansion of graphite lattice and changes in the sample thickness. Initially the rather smooth graphite surface (Figure 3a) becomes visibly wrinkled while the surface color remains unchanged (Figure 3b). Observation of the wrinkled surface of stage‐2 GIC is also in agreement with the Daumas‐Hérold theory, which assumes that the intercalate is occupying all graphite interlayer spaces as periodically occurring clusters.[^34^, ^35^] Further observations reveal rather strong and quick changes of morphology at the top of voltage cycle, followed by visually unchanging surface at the low voltage part of the cycle.

Rapid formation of stage‐1 GIC from the stage‐2 GIC surface (according to XRD data) is observed as the appearance of spots with a size ~10–20 μm with colors ranging from brown to yellow, with the strongest changes observed during the top voltage plateau. Very few changes are observed during the low voltage part of the cycle when stage‐1 GIC transforms into PGO, and differently colored clusters only become somewhat darker (Figure 3c and d). The process is repeated periodically with new colored spots added at the top of the voltage cycle until the whole surface is covered by PGO clusters (Figure 3e and f). A full video showing the changes of morphology during oxidation of graphite into PGO correlated with the galvanostatic curve is given in the Supporting Information (Video S1).

It is important to note that PGO was studied in our experiments directly in H_2_SO_4_ solution, in contrast to the “standard” water‐washed GO obtained in bulk synthesis. The interlayer distance of PGO provided by d(001) value is significantly higher than the one observed usually for standard dry GO (~7 Å)[^15^, ^16^, ^21^, ^36^, ^37^] due to swelling in aqueous sulfuric acid. The thickness of one sulfuric acid layer can be deduced using the known lattice expansion of stage‐1 H_2_SO_4_‐GIC, which is ~4.6 Å larger compared to graphite.[^16^, ^19^] The PGO *c*‐lattice is about 7 Å larger than that of standard dry (water‐washed) GO indicating that more than one layer of intercalate is confined between the layers of PGO.

**Figure 4:**
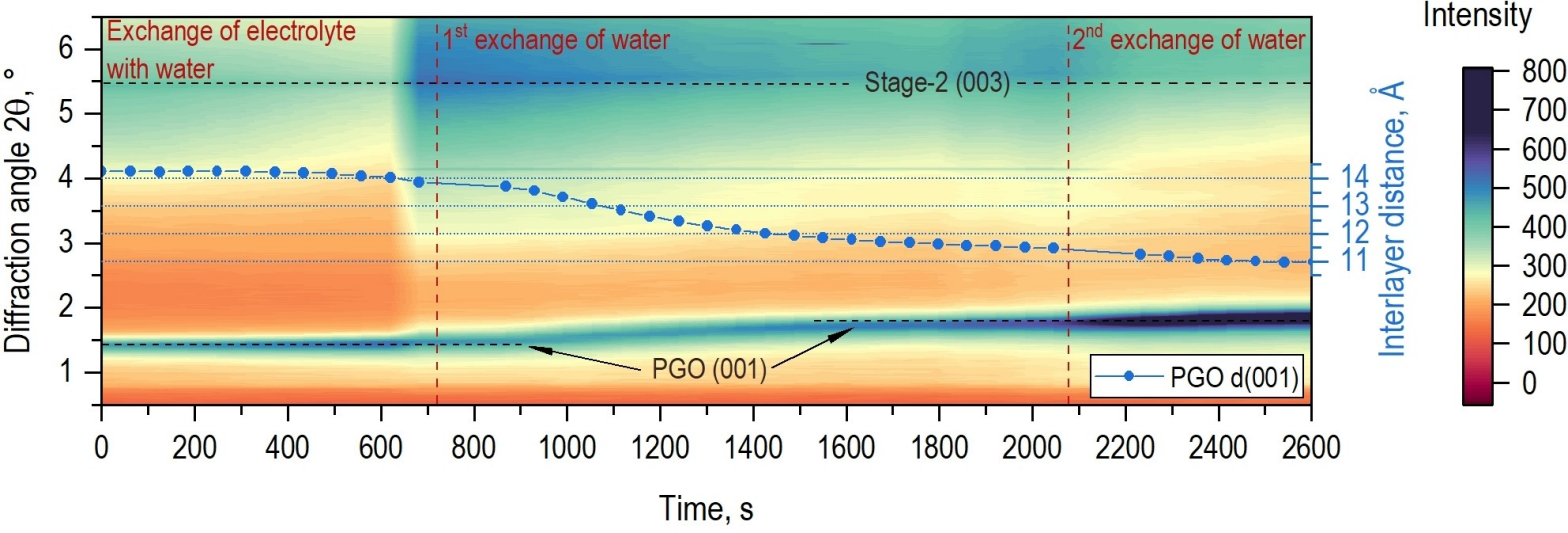
In situ synchrotron radiation XRD investigation of interlayer spacing changes in PGO caused by water‐washing.

The transformation of PGO into “standard” GO during the water‐washing was studied using an in situ time‐resolved experiment presented in Figure 4. The sample of PGO obtained at the end of the cycling experiment shown above (Figure 1) was water‐washed by replacing the electrolyte with deionized water inside the reactor while monitoring the structural changes by XRD. The interlayer distance of PGO decreased from 14.2 Å to ~11 Å after 3 water‐washing cycles. This value of d(001) is close to the water swollen state of standard water‐washed GO typically found after bulk synthesis.[^38^, ^39^] The interlayer spacing of GO immersed in water is about ~5 Å larger as compared to dry GO suggesting that at least two layers of water molecules (considering a thickness of approximately 2.5 Å for one layer) are present between the washed GO layers.^[40]^ The difference between d(001) values of PGO and GO in swollen state is then about 3.2 Å and can be explained by replacement of a thicker layer of H_2_SO_4_ intercalated in PGO with pure water in water‐immersed GO.

Additional information about the nature of oscillations can be provided by the analysis of bulk synthesis data. Well‐defined potential oscillations were found for experiments performed using 11 M H_2_SO_4_ but not in 13 M H_2_SO_4_ (Figure S1). This result is in agreement with earlier published studies of bulk electrochemical GO synthesis, which report that increasing sulfuric acid concentration results in a decrease of the overall oscillation number, the oscillation frequency, and the oscillation amplitude.^[24]^ The existence of oscillations in relatively diluted H_2_SO_4_ solutions and the absence of oscillations in a more concentrated acid point out the balance between amounts of bisulfate anions and water as one of the main conditions for appearance of oscillations.

**Figure 5:**
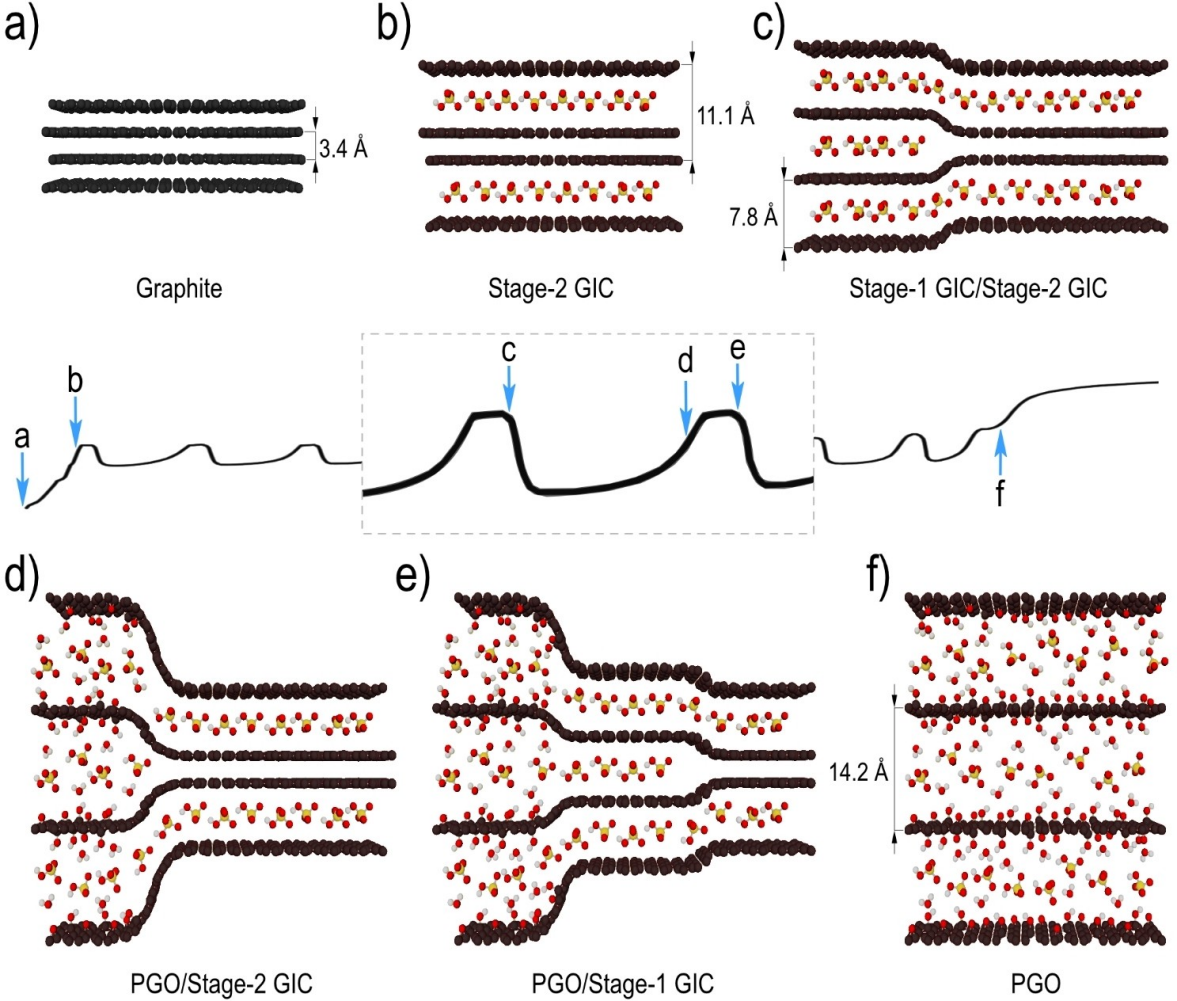
The Scheme illustrating mechanism of GO formation through the periodic structural transformations observed during electrochemical oxidation of graphite. The panels show all crystalline phases observed in the synthesis and parts of voltage curve prior to oscillations, fragment with one full cycle and the part in the end of oscillations. The structures shown in the panels a–f) are then correlated to certain points in the voltage curve relevant to the discussion provided in the text.

In situ synchrotron radiation XRD experiments presented in our study reveal new insights into the mechanism of electrochemical GO formation. The overall reaction pathway can be modeled as follows (Figure 5). Oscillatory anodic oxidation of graphite (in a certain range of sulfuric acid concentrations) starts with the formation of pure stage‐2 GIC (Figure 5b). The oscillations of the working electrode potential start after complete conversion of graphite into stage‐2 GIC, corresponding to the first maximum of the voltage curve. Oscillations are caused by two repeating processes which lead to the conversion of all stage‐2 GIC into PGO. The first process is partial transformation of stage‐2 GIC into stage‐1 GIC which occurs at the top of the voltage cycle (higher voltage plateau) (Figure 5c). The lower voltage plateau is associated with the oxygen functionalization of graphene layers by reacting with water, resulting in the formation of PGO (Figure 5d). When all stage‐1 GIC formed in the first half of the cycle is fully converted to PGO, the voltage rises again until the next part of stage‐2 GIC starts to be transformed into stage‐1 GIC (Figure 5e). These two processes occur periodically one after another thus resulting in voltage oscillations and structural oscillations related to appearance and disappearance of stage‐1 GIC. The oscillations last until most of the stage‐2 GIC is converted into PGO (Figure 5f), then the voltage oscillations fade. Subsequently, the potential of the working electrode increases and the electrolysis of water on the surface of the working electrode and current collector begins to occur.

Therefore, the main reason why the transformation from stage‐2 GIC into PGO occurs not as a continuous change, but through oscillations is the competition between two reactions:

– Reaction 1 is stage‐2 GIC to stage‐1 GIC conversion, which occurs in 11 M H_2_SO_4_ only at higher voltage (~1.94 V).
– Reaction 2 is conversion of stage‐1 GIC into PGO which requires lower voltage of ~1.8 V.

Reaction 2 starts only when some stage‐1 GIC is produced in Reaction 1. As long as the amount of stage‐1 GIC increases, the rate of Reaction 2 increases as well until it starts to prevail and voltage decreases below the point required for Reaction 1. The voltage starts to increase again only when all stage‐1 GIC has been converted into PGO (Reaction 2). The cycle restarts when the voltage increases again to the level required for the formation of stage‐1 GIC (Reaction 1).

The existence of oscillations is limited to a certain range of concentrations^[24]^ with a relatively high concentration of water in 11 M H_2_SO_4_ and, correspondingly, a not very high concentration of bisulfate anions. Therefore, it is likely that oscillations are also related to some changes in the concentration of H_2_SO_4_ close to the graphite electrode surface. When the constant current is applied to the reactor, the concentration of acid is likely to drop locally due to the intercalation of sulfuric acid into graphite structure and the dilution of the acid solution (enrichment with water) close to the surface of the solid material occurs. Then the water starts to co‐intercalate and reacts with graphene layers yielding PGO. The reaction of water with graphene layers increases the concentration of H_2_SO_4_ locally close to the surface of the material and the cycle restarts. When the reaction is performed in a more concentrated 13 M H_2_SO_4_ (Figure S1), the potential required for the formation of the stage‐1 GIC is smaller, and a larger part of stage‐2 can be transformed into stage‐1 GIC before water starts to co‐intercalate. A lower amount of water also leads to a decrease in the oscillation amplitude, and with even lower water amounts, oscillations are not found.

## Conclusions

In situ study of GO synthesis by oscillating electrochemical oxidation of graphite in aqueous sulfuric acid was performed using the time‐resolved synchrotron radiation XRD inside a specially designed, capillary‐sized electrochemical reactor, revealing unusual periodic changes in the phase composition of the system. Using XRD we were able to monitor changes in the phase composition of the system, reveal the rapid evolution of intermediate phases and to correlate the structural changes with the oscillations of the working electrode potential under constant applied current.

Complete transformation of graphite into stage‐2 GIC was observed within the first and relatively rapid part of the reaction. Instead of a gradual transformation of stage‐2 GIC into PGO over the course of several hours, we found that an intermediate stage‐1 GIC phase appears and disappears periodically in the system, and the transition into PGO occurs by the reaction of water with this phase until it is completely consumed in each oscillation cycle. Periodic changes of sample structure were also confirmed using optical microscopy, which showed that formation of stage‐1 GIC (and further into PGO) occurs in differently colored, multiple regions of 10–20 μm in size. Each cycle adds to the number of reacted regions.

Unlike GO typically studied ex situ after water‐washing, we demonstrated the existence of “pristine GO” with a significantly different structure and studied it directly in sulfuric acid solution. In situ experiments revealed that PGO is intercalated by sulfuric acid, which gets replaced by water in the process of water‐washing. The change from a PGO structure swollen in sulfuric acid to water‐swollen GO results in a change of interlayer distance from ~14.2 to ~11.0 Å.

We suggest that the oscillations of the graphite electrode potential found in this system are related to positive and negative feedback between two reactions that occur at different applied potentials: the first is the intercalation of bisulfate anions into stage‐2 GIC, yielding stage‐1 GIC, and the second is the reaction of water with graphene planes of stage‐1 GIC phase, resulting in the formation of “pristine GO”. The first reaction requires a higher potential and occurs at the maximum of the potential wave (a higher potential plateau) while the second requires a lower potential insufficient for stage‐2 to stage‐1 GIC conversion and proceeds until all stage‐1 GIC is converted into PGO. The number of oscillations, the oscillation frequency, and the potential amplitude are strongly affected by the concentration of sulfuric acid (proportion between amount water and sulfuric acid in the solution).

Oscillating reactions with periodic changes in component concentrations in a liquid phase are rare but well known by now.[^30^, ^31^, ^41^] The reaction studied here has features similar to other oscillating reactions: non‐equilibrium conditions, a source of external energy provided by a constant electrical current, the presence of two competing reactions and periodic changes in the composition of the system. However, the periodic appearance and disappearance of the solid phase with a certain well‐defined structure found in our experiments seem to represent a completely new feature not found in “classical” oscillating reactions. Similar to graphite oxidation, other types of electrochemical oscillations have been reported previously, but mostly based only on observations of potential oscillations.[^42^, ^43^] Therefore, it can be anticipated that periodic changes in solid phase compositions of other materials may be found in the future.

## Supporting Information

The authors have cited additional references within the Supporting Information.[^44^, ^45^, ^46^, ^47^, ^48^, ^49^, ^50^, ^51^, ^52^]

## Conflict of Interests

The authors declare no conflict of interest.

1

## Supporting information

As a service to our authors and readers, this journal provides supporting information supplied by the authors. Such materials are peer reviewed and may be re‐organized for online delivery, but are not copy‐edited or typeset. Technical support issues arising from supporting information (other than missing files) should be addressed to the authors.

Supporting Information

Supporting Information

## Data Availability

The data that support the findings of this study are available from the corresponding author upon reasonable request.

## References

[1] G. Srinivas , Y. Zhu , R. Piner , N. Skipper , M. Ellerby , R. Ruoff , Carbon 2010, 48, 630–635.

[2] L. Chen , G. Shi , J. Shen , B. Peng , B. Zhang , Y. Wang , F. Bian , J. Wang , D. Li , Z. Qian , G. Xu , G. Liu , J. Zeng , L. Zhang , Y. Yang , G. Zhou , M. Wu , W. Jin , J. Li , H. Fang , Nature 2017, 550, 380–383. 28992630 10.1038/nature24044

[3] K. R. Bang , C. Kwon , H. Lee , S. Kim , E. S. Cho , ACS Nano 2023, 17, 10000–10009. 37196224 10.1021/acsnano.2c11975

[4] H. Liu , X. Huang , Y. Wang , B. Kuang , W. Li , Nat. Commun. 2024, 15, 164. 38167389 10.1038/s41467-023-44626-9 PMC10762124

[5] Z. F. Wu , P. Z. Sun , O. J. Wahab , Y. T. Tan , D. Barry , D. Periyanagounder , P. B. Pillai , Q. Dai , W. Q. Xiong , L. F. Vega , K. Lulla , S. J. Yuan , R. R. Nair , E. Daviddi , P. R. Unwin , A. K. Geim , M. Lozada-Hidalgo , Nat. Commun. 2023, 14, 7756. 38012200 10.1038/s41467-023-43637-w PMC10682477

[6] M. Seredych , J. A. Rossin , T. J. Bandosz , Carbon 2011, 49, 4392–4402.

[7] N. Boulanger , A. S. Kuzenkova , A. Iakunkov , A. Y. Romanchuk , A. L. Trigub , A. V. Egorov , S. Bauters , L. Amidani , M. Retegan , K. O. Kvashnina , S. N. Kalmykov , A. V. Talyzin , ACS Appl. Mater. Interfaces. 2020, 12, 45122–45135. 32902246 10.1021/acsami.0c11122 PMC7684581

[8] R. Futamura , T. Iiyama , T. Ueda , P. A. Bonnaud , F.-X. Coudert , A. Furuse , H. Tanaka , R. J. M. Pellenq , K. Kaneko , Nat. Commun. 2024, 15, 3585. 38678034 10.1038/s41467-024-47838-9 PMC11055881

[9] D.-H. Kim , M.-J. Kim , S.-Y. Kwak , J. Jeong , D. Choi , S. W. Choi , J. Ryu , K.-S. Kang , Nat. Commun. 2023, 14, 801. 36781854 10.1038/s41467-023-35941-2 PMC9925774

[10] K. Chen , X. Tang , B. Jia , C. Chao , Y. Wei , J. Hou , L. Dong , X. Deng , T.-H. Xiao , K. Goda , L. Guo , Nat. Mater. 2022, 21, 1121–1129. 35798946 10.1038/s41563-022-01292-4

[11] B. C. Brodie , Philos. Trans. R. Soc. London 1997, 149, 249–259.

[12] L. Staudenmaier , Ber. Dtsch. Chem. Ges. 1898, 31, 1481–1487.

[13] W. S. Hummers Jr. , R. E. Offeman , J. Am. Chem. Soc. 1958, 80, 1339–1339.

[14] D. C. Marcano , D. V. Kosynkin , J. M. Berlin , A. Sinitskii , Z. Sun , A. Slesarev , L. B. Alemany , W. Lu , J. M. Tour , ACS Nano 2010, 4, 4806–4814. 20731455 10.1021/nn1006368

[15] B. Gurzęda , N. Boulanger , M. R. V. Jørgensen , I. Kantor , A. V. Talyzin , Carbon 2024, 221, 118899.

[16] A. M. Dimiev , J. M. Tour , ACS Nano 2014, 8, 3060–3068. 24568241 10.1021/nn500606a

[17] N. I. Kovtyukhova , P. J. Ollivier , B. R. Martin , T. E. Mallouk , S. A. Chizhik , E. V. Buzaneva , A. D. Gorchinskiy , Chem. Mater. 1999, 11, 771–778.

[18] S. Eigler , M. Enzelberger-Heim , S. Grimm , P. Hofmann , W. Kroener , A. Geworski , C. Dotzer , M. Röckert , J. Xiao , C. Papp , O. Lytken , H.-P. Steinrück , P. Müller , A. Hirsch , Adv. Mater. 2013, 25, 3583–3587. 23703794 10.1002/adma.201300155

[19] J. Cao , P. He , M. A. Mohammed , X. Zhao , R. J. Young , B. Derby , I. A. Kinloch , R. A. W. Dryfe , J. Am. Chem. Soc. 2017, 139, 17446–17456. 29090921 10.1021/jacs.7b08515

[20] S. Pei , Q. Wei , K. Huang , H.-M. Cheng , W. Ren , Nat. Commun. 2018, 9, 145. 29321501 10.1038/s41467-017-02479-z PMC5762692

[21] Z. Tian , P. Yu , S. E. Lowe , A. G. Pandolfo , T. R. Gengenbach , K. M. Nairn , J. Song , X. Wang , Y. L. Zhong , D. Li , Carbon 2017, 112, 185–191.

[22] B. Gurzęda , T. Buchwald , M. Nocuń , A. Bąkowicz , P. Krawczyk , RSC Adv. 2017, 7, 19904–19911. 10.1039/c8ra90043d PMC9080685 35543966

[23] H. Krohn , F. Beck , R. Herrmann , Chem. Ing. Tech. 1982, 54, 530–531.

[24] F. Beck , J. Jiang , H. Krohn , J. Electroanal. Chem. 1995, 389, 161–165.

[25] B. Gurzęda , P. Florczak , M. Kempiński , B. Peplińska , P. Krawczyk , S. Jurga , Carbon 2016, 100, 540–545.

[26] B. Gurzęda , P. Krawczyk , Electrochim. Acta 2018, 267, 102–109.

[27] N. E. Sorokina , N. V. Maksimova , V. V. Avdeev , Inorg. Mater. 2001, 37, 360–365.

[28] B. D. L. Campéon , M. Akada , M. S. Ahmad , Y. Nishikawa , K. Gotoh , Y. Nishina , Carbon 2020, 158, 356–363.

[29] A. Harrach , J. Douglade , A. Métrot , Mater. Sci. Forum 1992, 91 (93), 689–694.

[30] A. N. Zaikin , A. M. Zhabotinsky , Nature 1970, 225, 535–537. 10.1038/225535b0 16056595

[31] W. C. Bray , J. Am. Chem. Soc. 1921, 43, 1262–1267.

[32] K. Kurin-Csörgei , I. R. Epstein , M. Orbán , Nature 2005, 433, 139–142. 15650734 10.1038/nature03214

[33] A. M. Dimiev , S. M. Bachilo , R. Saito , J. M. Tour , ACS Nano 2012, 6, 7842–7849. 22880798 10.1021/nn3020147

[34] N. Daumas , A. Hérold , C. R. Acad. Sci., Ser. C: Sci. Chim. 1969, 268, 373–375.

[35] E. M. Gavilán-Arriazu , O. A. Pinto , B. A. López de Mishima , D. E. Barraco , O. A. Oviedo , E. P. M. Leiva , Electrochem. Commun. 2018, 93, 133–137.

[36] Y. Zhu , S. Murali , W. Cai , X. Li , J. W. Suk , J. R. Potts , R. S. Ruoff , Adv. Mater. 2010, 22, 3906–3924. 20706983 10.1002/adma.201001068

[37] D. A. Dikin , S. Stankovich , E. J. Zimney , R. D. Piner , G. H. B. Dommett , G. Evmenenko , S. T. Nguyen , R. S. Ruoff , Nature 2007, 448, 457–460. 17653188 10.1038/nature06016

[38] A. V. Talyzin , V. L. Solozhenko , O. O. Kurakevych , T. Szabó , I. Dékány , A. Kurnosov , V. Dmitriev , Angew. Chem. Int. Ed. 2008, 47, 8268–8271. 10.1002/anie.200802860 18814163

[39] A. Iakunkov , N. Boulanger , A. Nordenström , A. V. Talyzin , Adv. Mater. Interfaces 2021, 8, 2100552.

[40] A. V. Talyzin , T. Hausmaninger , S. You , T. Szabó , Nanoscale 2014, 6, 272–281. 24189605 10.1039/c3nr04631a

[41] Y. Suchorski , M. Datler , I. Bespalov , J. Zeininger , M. Stöger-Pollach , J. Bernardi , H. Grönbeck , G. Rupprechter , Nat. Commun. 2018, 9, 600. 29426883 10.1038/s41467-018-03007-3 PMC5807506

[42] M. T. M. Koper , J. Chem. Soc. Faraday Trans. 1998, 94, 1369–1378.

[43] D. Li , Y. Sun , Z. Yang , L. Gu , Y. Chen , H. Zhou , Joule 2018, 2, 1265–1277.

[44] A. Fitch , C. Dejoie , E. Covacci , G. Confalonieri , O. Grendal , L. Claustre , P. Guillou , J. Kieffer , W. de Nolf , S. Petitdemange , M. Ruat , Y. Watier , J. Synchrotron Radiat. 2023, 30, 1003–1012. 37462688 10.1107/S1600577523004915 PMC10481261

[45] G. Ashiotis , A. Deschildre , Z. Nawaz , J. P. Wright , D. Karkoulis , F. E. Picca , J. Kieffer , J. Appl. Crystallogr. 2015, 48, 510–519. 25844080 10.1107/S1600576715004306 PMC4379438

[46] M. Inagaki , N. Iwashita , E. Kouno , Carbon 1990, 28, 49–55.

[47] A. S. Kuzenkova , A. Y. Romanchuk , A. L. Trigub , K. I. Maslakov , A. V. Egorov , L. Amidani , C. Kittrell , K. O. Kvashnina , J. M. Tour , A. V. Talyzin , S. N. Kalmykov , Carbon 2020, 158, 291–302.

[48] P. Feicht , R. Siegel , H. Thurn , J. W. Neubauer , M. Seuss , T. Szabó , A. V. Talyzin , C. E. Halbig , S. Eigler , D. A. Kunz , A. Fery , G. Papastavrou , J. Senker , J. Breu , Carbon 2017, 114, 700–705.

[49] Z. Wang , Q. Yao , C. Neumann , F. Börrnert , J. Renner , U. Kaiser , A. Turchanin , H. J. W. Zandvliet , S. Eigler , Angew. Chem. Int. Ed. 2020, 59, 13657–13662. 10.1002/anie.202004005 PMC7496721 32315109

[50] K. H. Thebo , X. Qian , Q. Zhang , L. Chen , H.-M. Cheng , W. Ren , Nat. Commun. 2018, 9, 1486. 29662053 10.1038/s41467-018-03919-0 PMC5902455

[51] T. R. Gengenbach , G. H. Major , M. R. Linford , C. D. Easton , J. Vac. Sci. Technol. A 2021, 39, 013204.

[52] F. Kang , Y. Leng , T.-Y. Zhang , J. Phys. Chem. Solids 1996, 57, 889–892.

