# Supplementary material for: Oscillating Structural Transformations in the Electrochemical Synthesis of Graphene Oxide from Graphite
Source: Angew Chem Int Ed Engl. 2024 Oct 18;63(51):e202411673. doi: 10.1002/anie.202411673 (PMC11627124; doi:10.1002/anie.202411673)
Supplement: Supplementary file 1 — Supporting Information [file ANIE-63-e202411673-s001.pdf]

Angewandte Chemie
Eine Zeitschrift der Gesellschaft Deutscher Chemiker

Supporting Information

Oscillating Structural Transformations in the Electrochemical Synthesis of Graphene Oxide from Graphite

B. Gurzęda, P. Jeżowski, N. Boulanger, A. V. Talyzin**

Supporting Information

Oscillating Structural Transformations in the Electrochemical Synthesis of Graphene Oxide from Graphite

B. Gurzęda, P. Jeżowski, N. Boulanger, and A. V. Talyzin**

Table of Contents

1.	**Experimental section**	**3**
1.1.	**In situ electrochemical graphite oxidation**	**3**
1.2.	**Bulk graphite electrochemical oxidation**	**3**
1.3.	**In situ optical microscope observations of graphite transformation into PGO**	**4**
1.4.	**Characterization**	**4**
2.	**Bulk electrochemical synthesis of GO**	**4**
	**Figure S1**	**5**
	**Table S1**	**6**
3.	**XPS analysis of GO prepared in bulk experiment**	**6**
	**Figure S2**	**7**
	**Table S2**	**7**
	**Figure S3**	**8**
	**Figure S4**	**8**
	**Figure S5**	**9**
	**Figure S6**	**10**
	**Figure S7**	**11**
	**Figure S8**	**11**
	**Figure S9**	**12**
	**Figure S10**	**12**
	**Figure S11**	**13**
	**Figure S12**	**14**
	**References**	**14**

1. Experimental section

1.1. In situ electrochemical graphite oxidation

A glass Pasteur pipette with 1.1 mm inner diameter and 0.2 mm wall thickness was used as a body of capillary size electrochemical reactor (Figure S8a). A platinum wire (99.9% Pt, 1 mm diameter, Goodfellow) playing the role of current collector for working electrode (~15 mm in length) was placed inside of the bottom part of the reactor and sealed with PTFE tape and parafilm. Graphite natural flakes (99% C, -100 mesh (≥80%), Sigma-Aldrich) were placed on the top of platinum wire inside the capillary reactor and held on place by piece of glass microfiber filter (~1.5 x 1.5 mm, thickness 0.26 mm, pore size 1.6 µm, Whatman). Micro-electrochemical reactor was filled with 11 M H_2SO_4 (95-97%, Merck) and platinum wire playing a role of counter and reference electrode was immersed in the wider top part of the reactor to avoid hydrogen accumulation inside the capillary which could break an electrolyte contact between the working and counter electrode. The electrochemical cell was closed using polypropylene plastic cover to reduce electrolyte evaporation (Figure S8b). Graphite (~280 µg) was anodically oxidized using a galvanostatic technique. A constant current of 40 µA (~143 mA g^{-1}) was passed through the working electrode, until the voltage oscillations faded down.

1.2. Bulk graphite electrochemical oxidation

Bulk graphite anodic oxidation was performed for two graphite samples: graphite flakes used in in situ studies and natural graphite with flake size >300 µm (99.95% C, ProGraphiteShop). The working electrode consisted of 50 mg of graphite enclosed between two platinum gauzes (22 x 22 mm, purity 99.9% 120 mesh, Goodfellow) held together by specially designed 3D printed polypropylene (PP) frames (Figure S9). For graphite with flake size -100 mesh a glass microfiber membrane was additionally used to prevent graphite from falling out through platinum gauze openings. The working electrode thus prepared was placed in the 25 mL beaker containing ~20 mL of 11 or 13 M H_2SO_4. Platinum wire was used as a counter electrode and Ag/Ag_2SO_4/1 M H_2SO_4 was used as a reference electrode (Figure S10). A galvanostatic technique was used with a current density of 30 mA g^{-1}. The process was cut off when the working electrode reached charge density equal to 4500 C g^{-1}. Prepared electrochemical graphite oxide (GO) materials were washed firstly with 10% HCl (37%, VWR) and next with deionized water until filtrate became neutral and freeze-dried. GO synthesized in 11 and 13 M H_2SO_4 using graphite with flake size -100 mesh were named 11S-100 and 13S-100,

respectively, and GO synthesized in 11 and 13 M H_2SO_4 using graphite with flake size >300 μm were named 11S-300 and 13S-300, respectively.

1.3. In situ optical microscope observations of graphite transformation into PGO

Highly oriented pyrolytic graphite (~500 x 500 μm) was electrochemically oxidized in 3D printed polyethylene (PE) reactor (Figure S11) using galvanostatic technique (~120 mA g^{-1}). A platinum foil (99.9% Pt, 0.1 mm thickness, Goodfellow) was used as current collector and counter electrode. Graphite was held on platinum foil by thin glass slide. A second piece of platinum foil played the role of counter and reference electrode. The process of graphite transformation into PGO was monitored for 5 hours.

1.4. Characterization

Electrochemical oxidation of graphite in aqueous sulfuric acid was carried out using potentiostat/galvanostat Autolab PGSTAT204. The synchrotron radiation XRD data were recorded at beamline ID22 at the European Synchrotron Radiation Facility (ESRF) using transmission geometry[1] and a Perkin Elmer XRD 1611CP3 detector positioned at the distance of 1400 mm from the electrochemical reactor. The X-ray wavelength was calibrated as 0.3543516 Å (35 keV) via a NIST standard 640c Si powder. The XRD patterns were collected during time-resolved experiments with typical parameters as following: 5 frames (0.5s per frame) and 45 sec waiting time. Azimuthal integration was carried out using the PyFAI library[2]. Bulk electrochemical synthesis of GO was performed using potentiostat/galvanostat Ivium-n-Stat with dModule(1A/20V). XRD patterns of GO prepared in bulk experiments were recorded using a Panalytical X'pert X-ray diffractometer with Cu Kα radiation. Cu Kα average (λ=1.5418 Å) was used for the calculation of d-spacings of the low angle reflections. XRD spectrum was recorded using a Kratos Axis Ultra electron spectrometer equipped with a delay line detector. A monochromatic 140 W Al Kα source was used as the excitation source. The binding energy scale was adjusted with respect to the C1s line of aliphatic carbon, set at 285.0 eV.

2. Bulk electrochemical synthesis of GO

Experiments with bulk electrochemical graphite oxide (GO) synthesis (Figure 1S) confirm that the number of oscillations, the oscillation frequency, and amplitude decrease if the reaction is performed with less concentrated (more water -rich) H_2SO_4.[3] Figure S1 also shows that graphite flake size affects oscillations in several ways.

It is also known that water concentration affects graphite intercalation by sulphuric acid. Higher water amount decreases the concentration of intercalate and higher potential is needed to begin the H_2SO_4 intercalation as well as to transform stage-2 into stage-1GIC.[4] Therefore, when the graphite is electrochemically oxidized in 11 M H_2SO_4 higher water concentration causes the transformation of only a small part of stage-2 into stage-1 GIC before water starts to co-intercalate and reacts with graphene layers yielding GO. When 13 M H_2SO_4 is used, the potential of stage-1 GIC formation decreases, and larger part of stage-2 can be transformed into stage-1 GIC before water starts to co-intercalate. Consequently, more charge is needed to transform GIC into GO and the frequency of potential oscillations degreases significantly. At the same time, due to the lower concentration of water in 13 M H_2SO_4 as compared to 11 M solution, the potential needed for water co-intercalation and the formation of oxygen functional groups increases which causes the decrease in the oscillation amplitude.

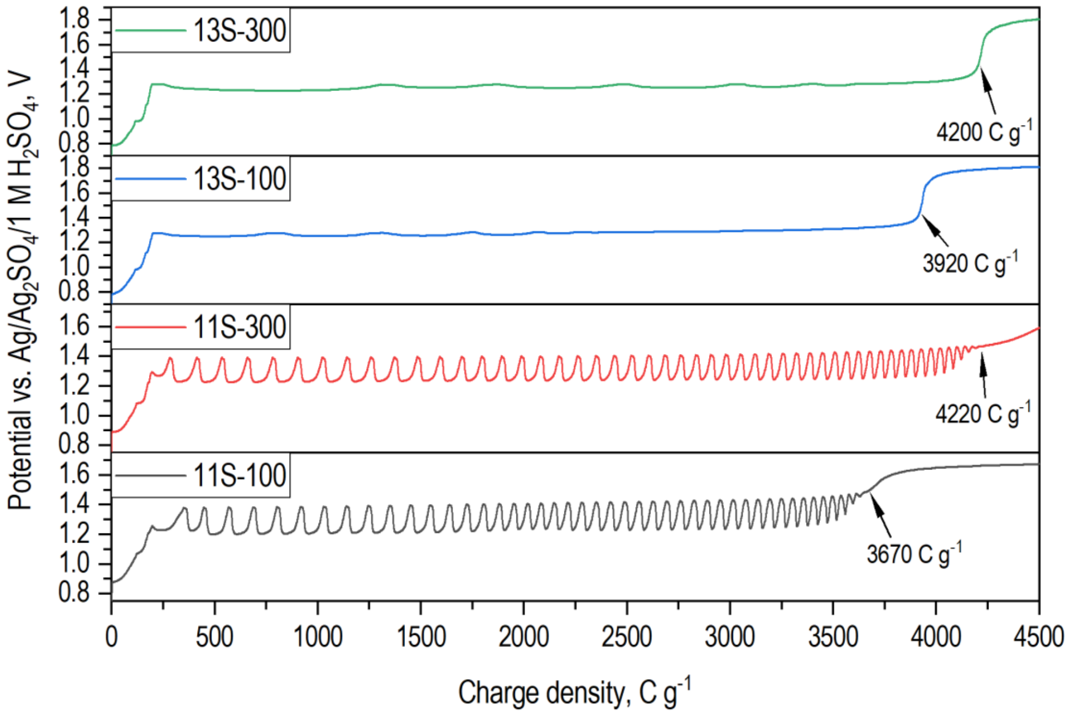

Figure S1. Galvanostatic curves recorded during the bulk synthesis of electrochemical GO. The synthesis was performed using two different sulfuric acid concentrations and two different graphite flake sizes. GO synthesized in 11 and 13 M H_2SO_4 using graphite with flake size -100 mesh were named 11S-100 and 13S-100, respectively, and GO synthesized in 11 and 13 M H_2SO_4 using graphite with flake size >300 μm were named 11S-300 and 13S-300, respectively.

The XRD analysis of GO samples prepared in bulk experiments shows that the electrochemical transformation of graphite into GO is also somewhat affected by the precursor graphite particle size. (Figures S1 and S12). The oscillating reaction takes longer time for complete oxidation of graphite into GO for sample with larger flake size. The GO formation efficiency correlates with the overall charge density consumed for the graphite anodic oxidation (Table S1).

Table S1. Overall charge densities for GO samples synthesized in bulk electrochemical experiments.

**GO sample**	**Overall charge densities, C g-1**
**11S-100**	3670
**11S-300**	4220
**13S-100**	3920
**13S-300**	4200

3. XPS analysis of GO prepared in bulk experiment

The chemical composition of GO prepared by bulk electrochemical oxidation of graphite with flake size -100 mesh in 13 M H_2SO_4 was investigated by X-ray photoelectron spectroscopy (XPS) analysis (Figure S2). Survey spectra of 13S-100 prepared in bulk GO synthesis (Figure S2a) shows that electrochemical GO composed mostly of carbon and oxygen atoms. Calculated C/O ratio is equal to 3.4 and is notably lower as compared to the GO synthesized using chemical methods (typically in the range of 2-2.7).[5-7] The deconvoluted high-resolution spectrum of C 1s region provides valuable information about the type of oxygen function groups bonded to the graphene layers (Figure S2b). As could be expected, the C 1s spectrum is typical for standard types of GO.[8-12] Electrochemically prepared GO shows mostly hydroxyl and epoxy groups as evidenced by component at 286.9 eV in the C1s spectrum.[7, 13] Low concentration of carbonyl and carboxyl groups is also detected at 288.3 and 289.3 eV, respectively. The last signal at 291.4 eV corresponds to π-π* satellites originating from unoxidized fraction of graphite structure (Table S2).

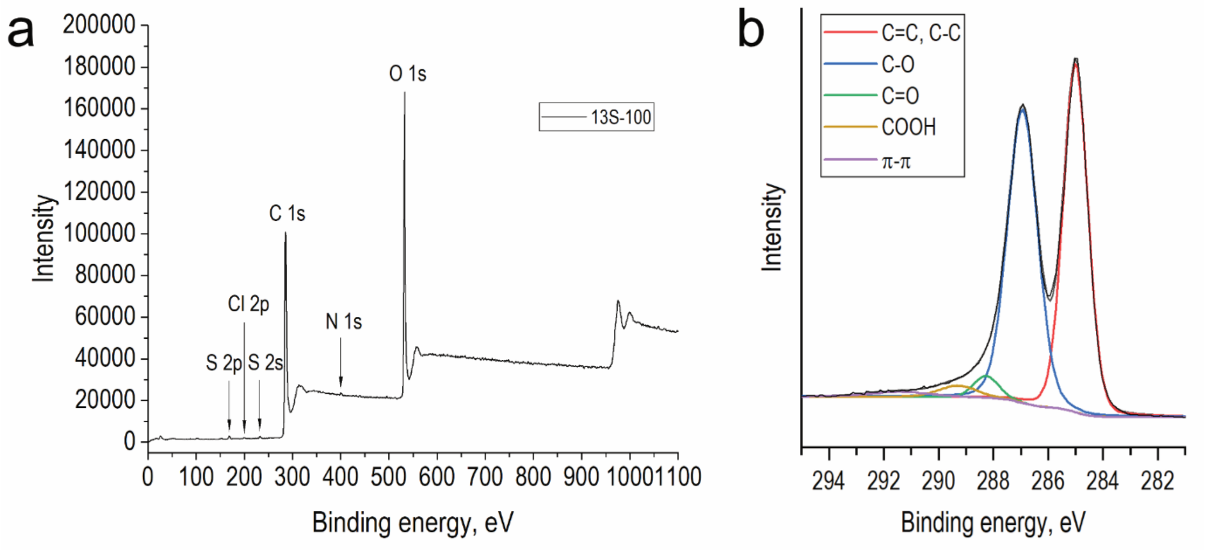

Figure S2. a) Survey XPS spectrum and b) high resolution C 1s spectrum recorded for GO synthesized by electrochemical oxidation of graphite with flake size -100 mesh in 13 M H_2SO_4.

Table S2. Results of the C 1s high-resolution XPS spectrum and impurities content of for GO synthesized by electrochemical oxidation of graphite with flake size -100 mesh in 13 M H_2SO_4.

	**13S-100**	
	**Area, %**	**Position, eV:**
**C=C, C-C**	45.60%	285.0
**C-O-C, C-OH**	46.68%	286.9
**C=O**	2.64%	288.3
**COO**	3.40%	289.3
**π-π satellite**	1.68%	291.4
**C/O ratio**	3.42	
**Impurities, At%**		
**S**	0.52%	
**N**	0.24%	
**Cl**	0.10%	

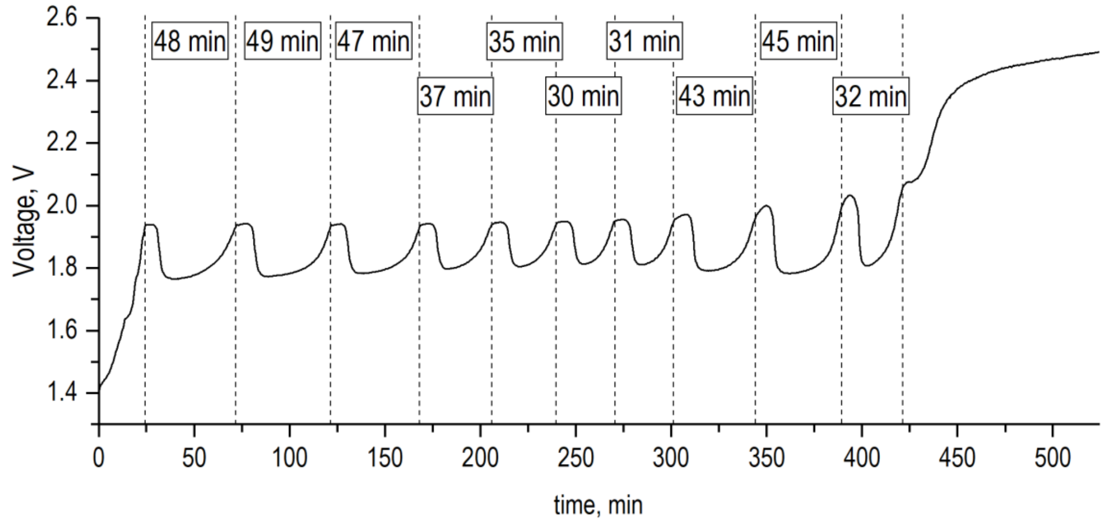

Figure S3. Galvanostatic curves recorded during in situ time-resolved XRD study of electrochemical graphite oxidation. The galvanostatic curve is plotted as a function of time showing the oscillation periods.

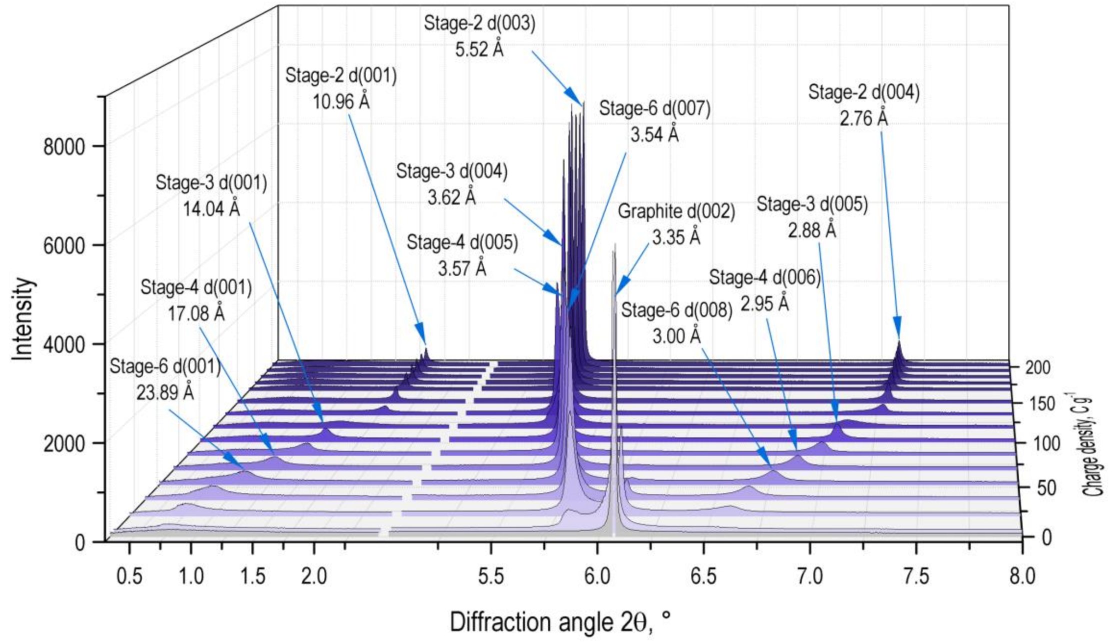

Figure S4. Synchrotron radiation XRD patterns recorded during the beginning of graphite oxidation showing the changes in graphite intercalation compound (GIC) staging until pure stage-2 GIC is formed. Interlayer d-spacings 23.89, 17.08, 14.04, and 10.96 Å are assigned to stage-6, stage-4, stage-3 and stage-2 GIC, respectively.[4, 14-15]

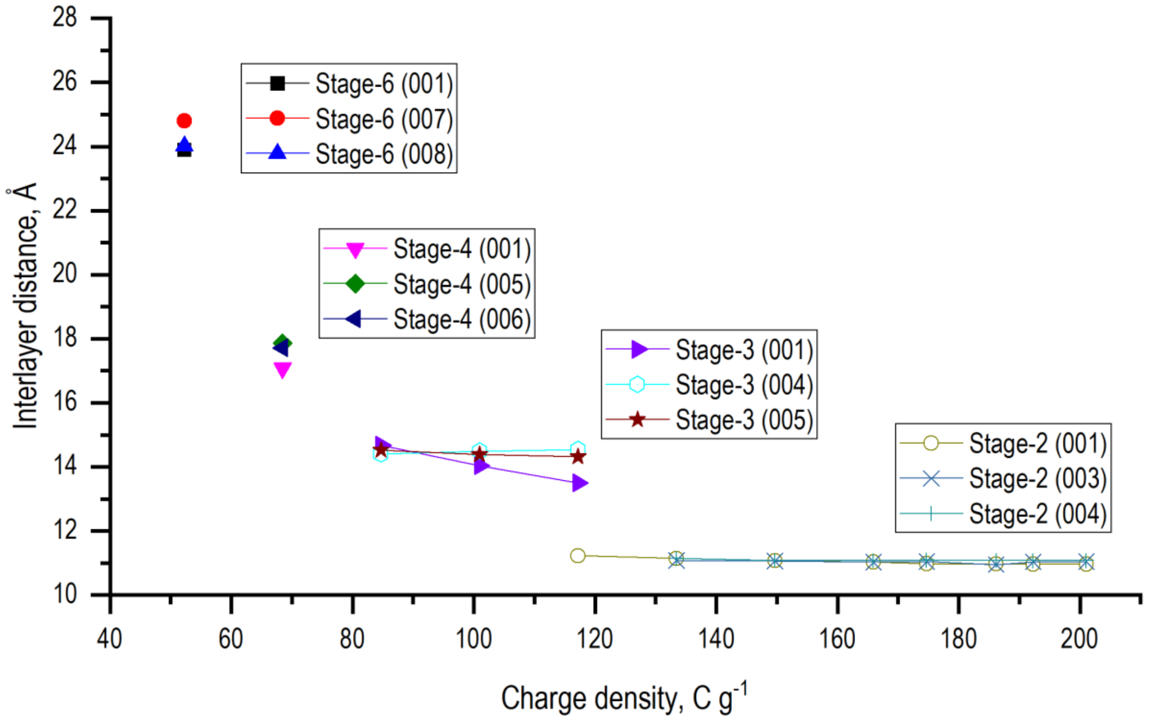

Figure S5. Interlayer spacing calculated based on the XRD patterns presented in Figure S4. Graph shows the changes in GIC staging during the electrochemical stage-2 GIC formation.

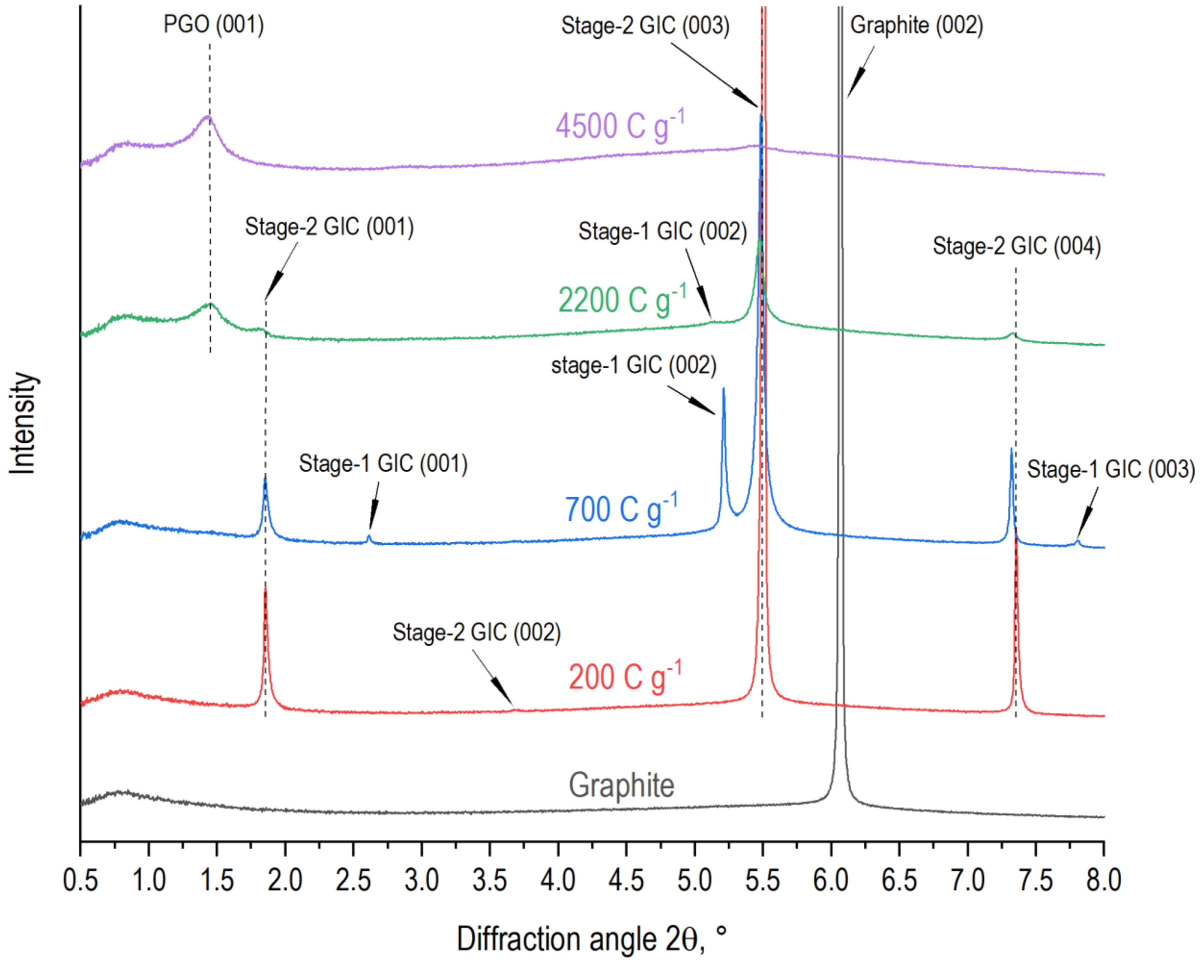

Figure S6. Chosen XRD patterns recorded during in situ synchrotron radiation XRD analysis showing the intermediate products formed during the electrochemical GO synthesis.

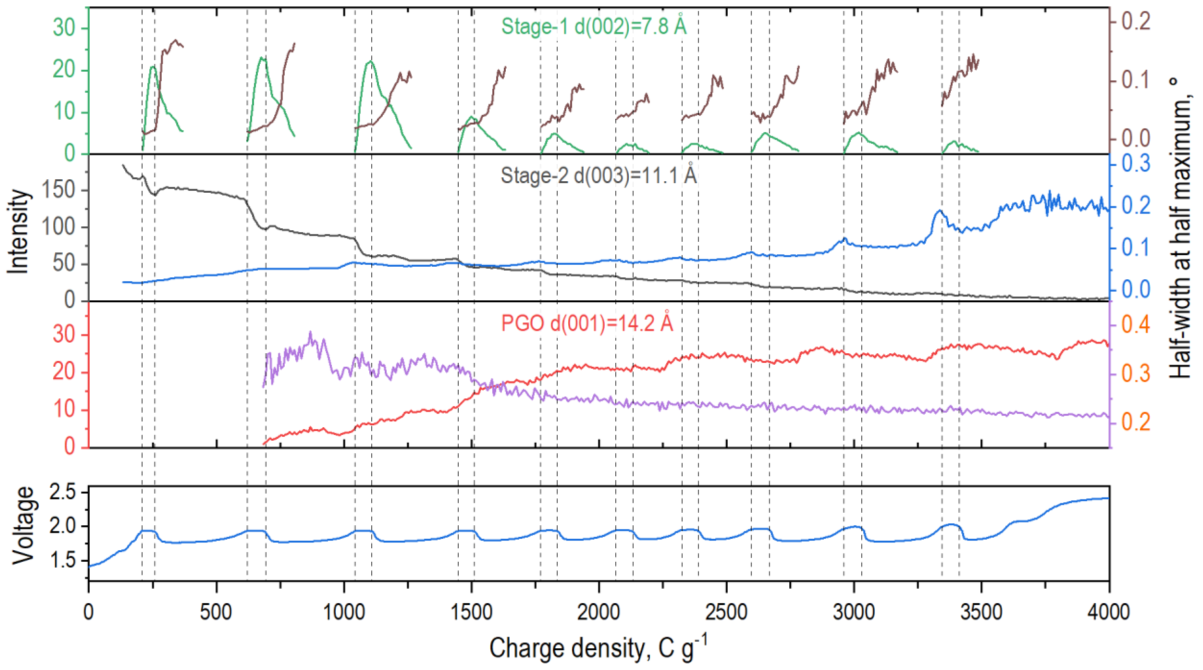

Figure S7. The changes in the intensities and half-widths at half maximum of signals assigned to stage-1 GIC, stage-2 GIC and PGO caused by the electrochemical graphite oxidation.

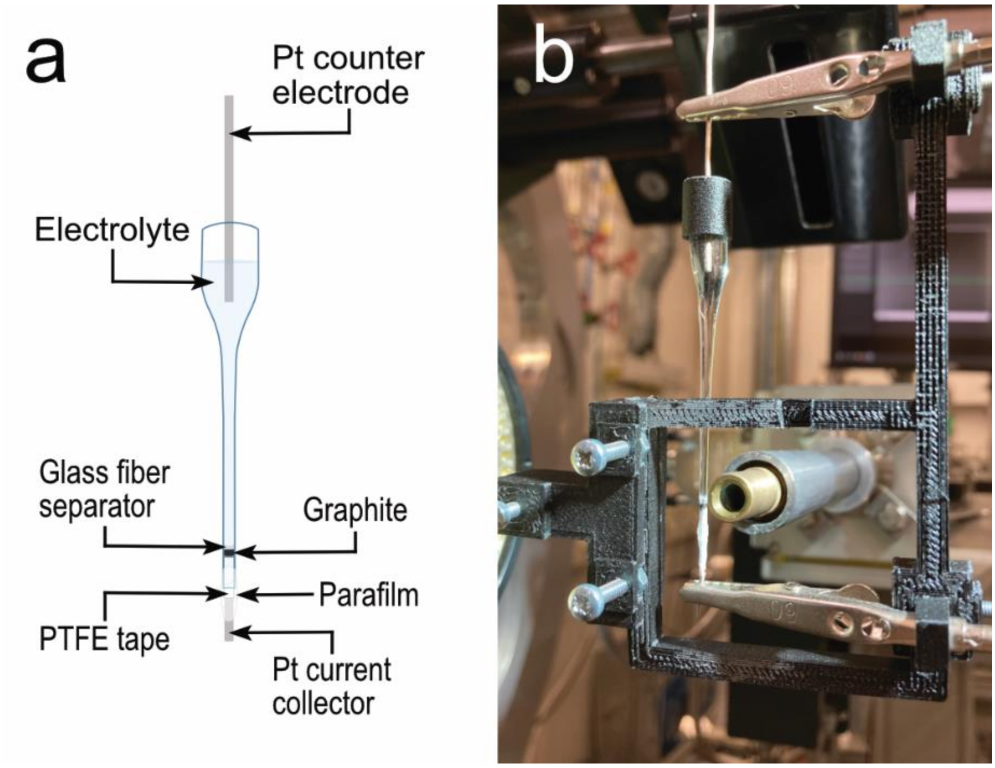

Figure S8. a) Scheme of the micro-electrochemical reactor. b) Photo of the installed reactor aligned in synchrotron radiation beam and connected to the potentiostat-galvanostat.

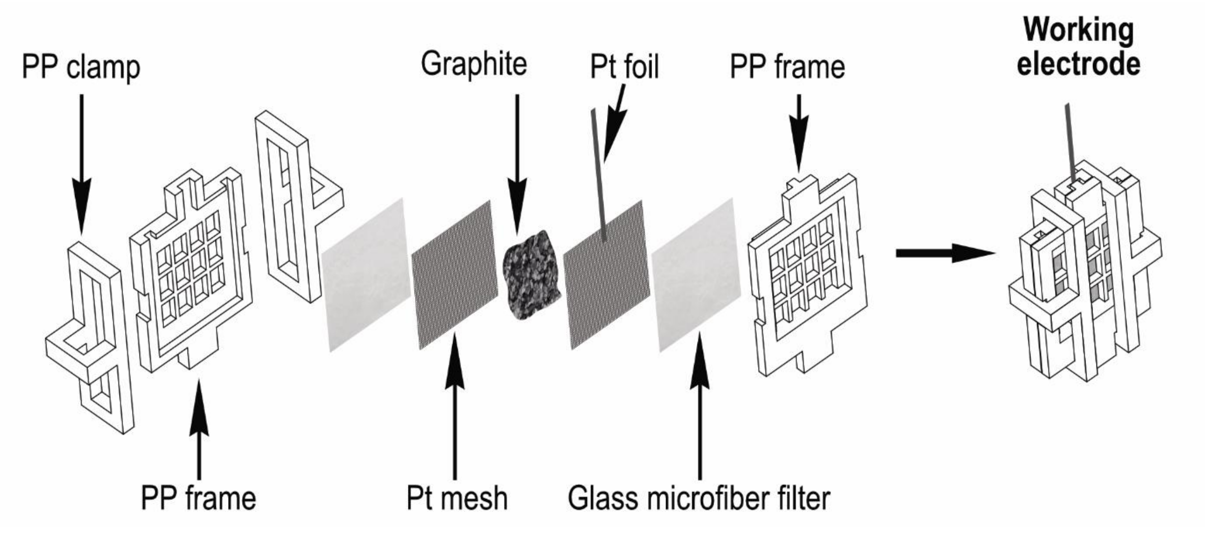

Figure S9. Scheme illustrating the construction of the working electrode used for bulk electrochemical GO synthesis.

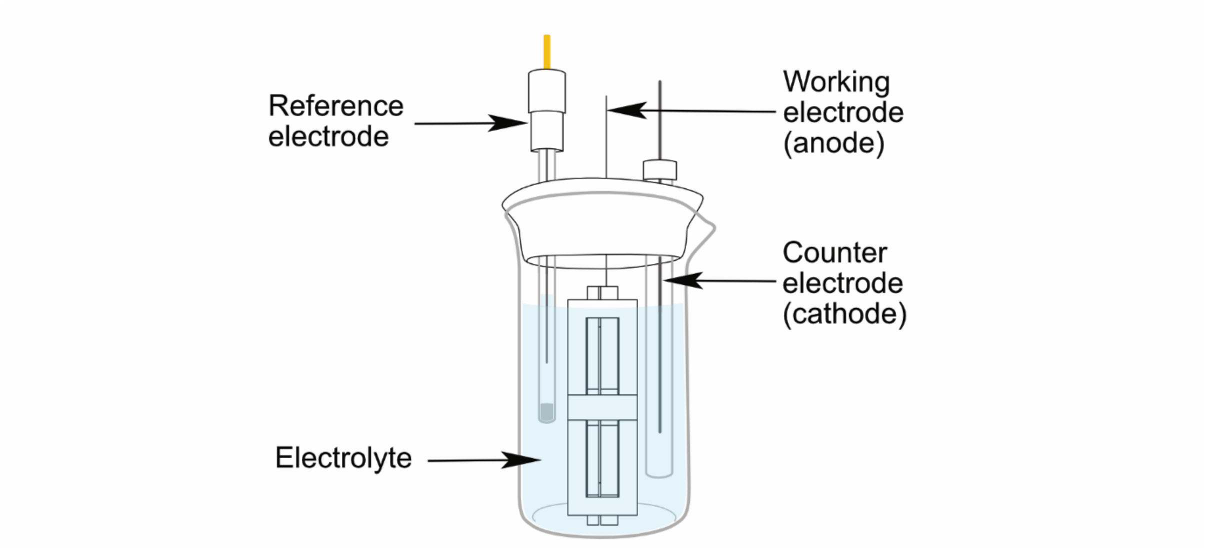

Figure S10. Scheme illustrating a three-electrode system used for bulk electrochemical GO synthesis. The working electrode prepared as shown in Figure S9 is immersed in electrolyte. Platinum wire is used as a counter electrode and is placed in glass tube. Ag/Ag_2SO_4/1 M H_2SO_4 electrode plays the role of reference electrode.

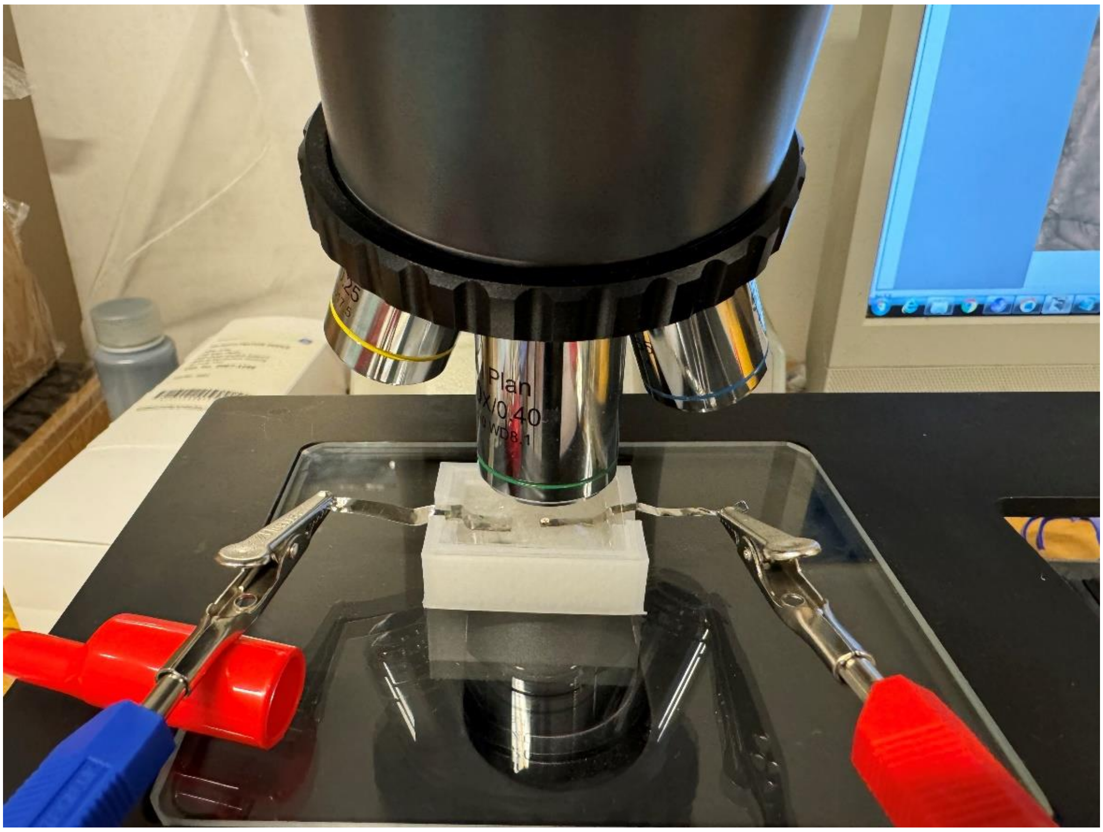

Figure S11. Photo of 3D printed PE reactor used for graphite electrochemical oxidation in 11 M H_2SO_4.

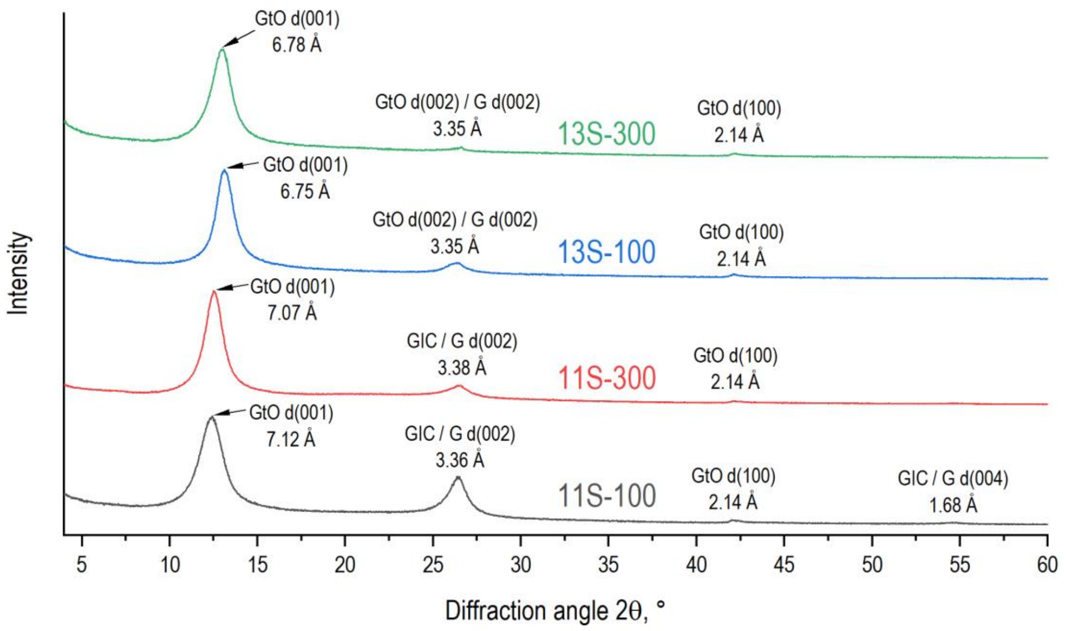

Figure S12. XRD patterns recorded for the GO samples synthesized in the bulk electrochemical experiments. GO synthesized in 11 and 13 M H_2SO_4 using graphite with flake size -100 mesh were named 11S-100 and 13S-100, respectively, and GO synthesized in 11 and 13 M H_2SO_4 using graphite with flake size >300 µm were named 11S-300 and 13S-300, respectively.

References

[1] A. Fitch, C. Dejoie, E. Covacci, G. Confalonieri, O. Grendal, L. Claustre, P. Guillou, J. Kieffer, W. de Nolf, S. Petitdemange, M. Ruat, Y. Watier, *Journal of Synchrotron Radiation* **2023**, *30*, 1003-1012.

[2] G. Ashiotis, A. Deschildre, Z. Nawaz, J. P. Wright, D. Karkoulis, F. E. Picca, J. Kieffer, *J. Appl. Crystallogr.* **2015**, *48*, 510-519.

[3] F. Beck, J. Jiang, H. Krohn, *J. Electroanal. Chem.* **1995**, *389*, 161-165.

[4] M. Inagaki, N. Iwashita, E. Kouno, *Carbon* **1990**, *28*, 49-55.

[5] A. S. Kuzenkova, A. Y. Romanchuk, A. L. Trigub, K. I. Maslakov, A. V. Egorov, L. Amidani, C. Kittrell, K. O. Kvashnina, J. M. Tour, A. V. Talyzin, S. N. Kalmykov, *Carbon* **2020**, *158*, 291-302.

[6] P. Feicht, R. Siegel, H. Thurn, J. W. Neubauer, M. Seuss, T. Szabó, A. V. Talyzin, C. E. Halbig, S. Eigler, D. A. Kunz, A. Fery, G. Papastavrou, J. Senker, J. Breu, *Carbon* **2017**, *114*, 700-705.

[7] Z. Wang, Q. Yao, C. Neumann, F. Börrnert, J. Renner, U. Kaiser, A. Turchanin, H. J. W. Zandvliet, S. Eigler, *Angew. Chem. Int. Ed.* **2020**, *59*, 13657-13662.

[8] J. Cao, P. He, M. A. Mohammed, X. Zhao, R. J. Young, B. Derby, I. A. Kinloch, R. A. W. Dryfe, *J. Am. Chem. Soc.* **2017**, *139*, 17446-17456.

[9] S. Pei, Q. Wei, K. Huang, H.-M. Cheng, W. Ren, *Nat. Commun.* **2018**, *9*, 145.
[10] Z. Tian, P. Yu, S. E. Lowe, A. G. Pandolfo, T. R. Gengenbach, K. M. Nairn, J. Song, X. Wang, Y. L. Zhong, D. Li, *Carbon* **2017**, *112*, 185-191.
[11] D. C. Marcano, D. V. Kosynkin, J. M. Berlin, A. Sinitskii, Z. Sun, A. Slesarev, L. B. Alemany, W. Lu, J. M. Tour, *ACS Nano* **2010**, *4*, 4806-4814.
[12] K. H. Thebo, X. Qian, Q. Zhang, L. Chen, H.-M. Cheng, W. Ren, *Nat. Commun.* **2018**, *9*, 1486.
[13] T. R. Gengenbach, G. H. Major, M. R. Linford, C. D. Easton, *J. Vac. Sci. Technol. A* **2021**, *39*, 013204.
[14] A. M. Dimiev, S. M. Bachilo, R. Saito, J. M. Tour, *ACS Nano* **2012**, *6*, 7842-7849.
[15] F. Kang, Y. Leng, T.-Y. Zhang, *J. Phys. Chem. Solids* **1996**, *57*, 889-892.
